# Modeling geographical invasions of *Solenopsis invicta* influenced by land-use patterns

**DOI:** 10.1038/s41598-022-15399-w

**Published:** 2022-08-02

**Authors:** Chia-Hsien Lin, Yi-Huei Liu, Rong-Nan Huang, Chung-Chi Lin, Helen Kang-Huey Liu, Tzai-Hung Wen

**Affiliations:** 1 Department of Geography, National Taiwan University, No. 1, Sec. 4, Roosevelt Road, 10617 Taipei City, Taiwan, ROC; 2 Department of Health Promotion and Health Education, National Taiwan Normal University, No. 162, Sec. 1, Heping E. Road, Taipei City, 10610 Taiwan, ROC; 3 Department of Entomology, National Taiwan University, No. 1, Sec. 4, Roosevelt Road, Taipei City, 10617 Taiwan, ROC; 4 Department of Biology, National Changhua University of Education, No. 1, Jinde Road, Changhua City, 50007 Taiwan, ROC; 5 Department of Political Science, National Taiwan University, No. 1, Sec. 4, Roosevelt Road, Taipei City, 10617 Taiwan, ROC

**Keywords:** Biogeography, Invasive species

## Abstract

Research into geographical invasions of red imported fire ants (RIFAs) by anthropogenic disturbances has received much attention. However, little is known about how land-use change and the characteristics of roads with different land-use types are associated with the risk of RIFA successful invasion or remaining at the highest level of invasion (RIFA SIRH). Furthermore, it was often assumed in prior studies that the risk of RIFA SIRH had a linear association with the independent variables. However, a linear relationship may not reflect the actual circumstances. In this study, we applied linear and nonlinear approaches to assess how land-use types, distance from the nearest road, different land-use types, and spatial factors affect the risk of RIFA SIRH. The results showed that agricultural land, land for transportation usage, and areas that had undergone land-use change from 2014 to 2017 had greater odds of RIFA invasion than natural land cover. We also identified land for transportation usage and the area of land-use change from 2014 to 2017, had more than 60% of RIFA SIRH within 350 m and 150 m from the nearest road. This study provided important insights into RIFA invasions in an isolated island and the areas of control strategies implemented.

## Introduction

The red imported fire ant (RIFA), *Solenopsis invicta* Buren, is an invasive species in many countries. Similar to other exotic species, RIFAs harm local ecosystems. RIFAs have noted for displacing indigenous fire ant species, such as *Solenopsis geminate* in Texas^1^ and *Solenopsis richteri* Forel in Mississippi^2^ as well as other ant species in the U.S.^3^. In addition, RIFA reduces biodiversity by decreasing populations of animals^4–6^ and damaging plants^7,8^. Therefore, understanding the patterns of geographical invasion for RIFA is essential in implementing controls to conserve local ecosystems, which is especially crucial to isolated islands^9^.

The geographical invasion of RIFAs can be due to natural dispersals (e.g., nuptial flights and flooding) as well as anthropogenic disturbances. Among the latter, the characteristics of roads are particularly conducive to RIFA invasion and establishment. Kelly and Sellers showed that roadsides had higher densities of RIFA than cypress savannas in a 2007 field survey in North Carolinas^10^. Tschinkel conducted a survey to identify the distribution of RIFAs in northern Florida. Greatly disturbed road areas such as road margins or graded roads were observed to have frequent occurrences of monogynous forms of RIFA^11^. Stiles and Jones’s survey also showed that more disturbed roadsides may be associated with a higher density of RIFA mounds compared to those with fewer disturbances in South Carolina^12^. However, most RIFA studies were surveyed in a specific year. These survey studies may not capture temporal variations in roads and RIFA among years. Without quantifying RIFA among years, those studies could not evaluate the associations between geographical invasion of RIFAs and characteristics of roads. In addition, studies often applied linear regressions that assume that each independent variable (e.g., distance to the road) has the same relationship with the dependent variable (e.g., RIFA occurrence) among years. This assumption may not reflect the complex process of RIFA expansions and the characteristics of roads for meeting the actual situation.

Land-use change is another type of important anthropogenic process that could favor the initiation of nonindigenous species^13,14^. Sánchez-Ortiz et al. modeled the effects of land-use change on nonindigenous and indigenous species by applying more than 6,000 animal and plant species^15^. The findings showed that land-use change was associated with an increased number of nonindigenous species. Jesse et al. surveyed more than 100 reptile communities on two Caribbean islands, and the results also showed that human-impacted land areas were associated with increased numbers of nonindigenous species^16^. Nevertheless, few published works have investigated the effect of land-use change on RIFA.

**Figure 1:**
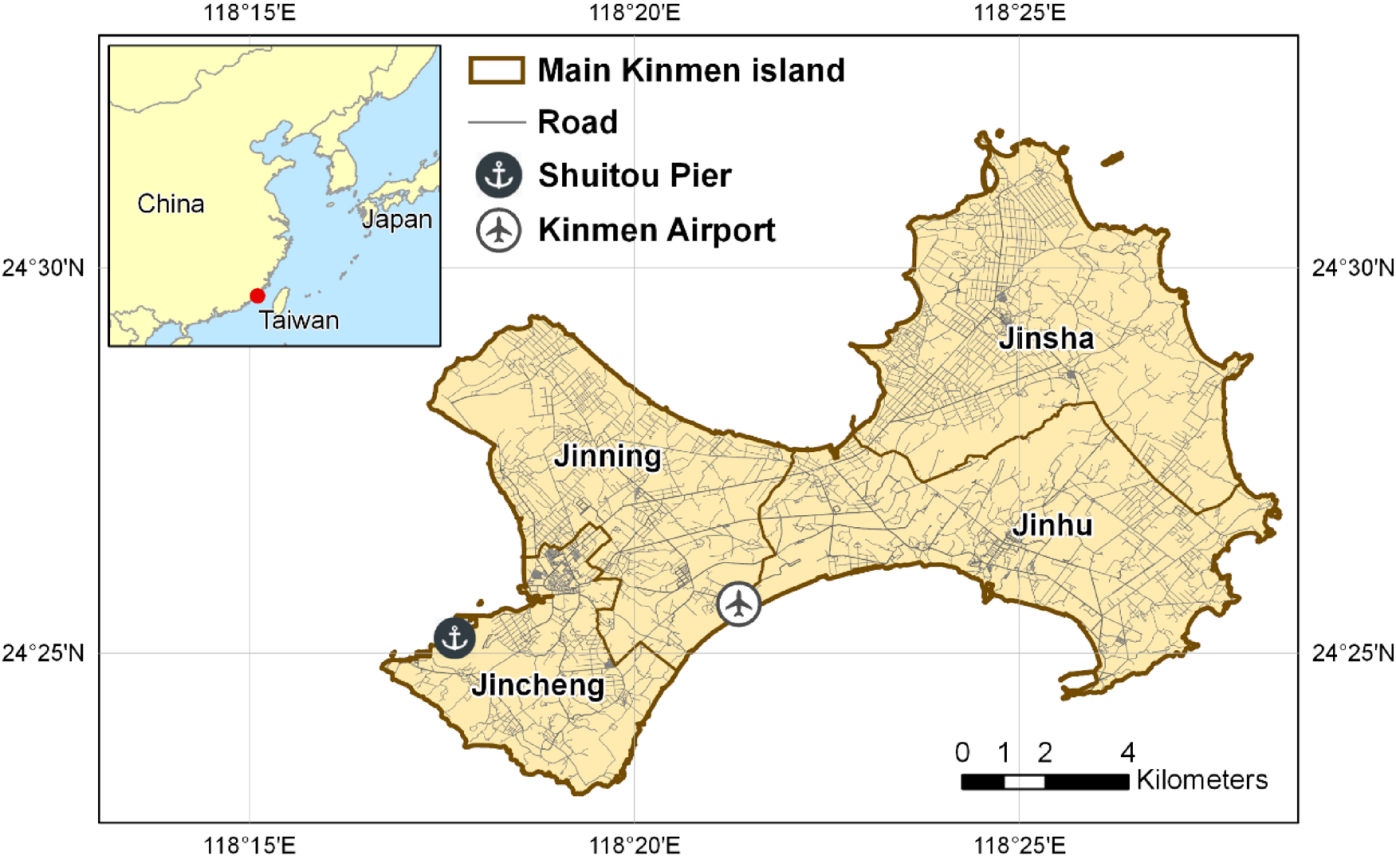
The location of four townships in the main Kinmen island, Taiwan. The red point in the index map is Kinman. This map is generated by ArcGIS 10.7^19^.

To understand the geographical expansions of RIFAs on an island, we conducted our study on the main Kinmen Island, Taiwan. Kinmen Island was chosen because by 2014, only small RIFA colonies had been detected in the northern part of Kinmen Island in Jinsha and Jinning townships (Fig. 1), but RIFA gradually expanded to the entire island by 2019. In addition, since 2015, the National RIFA Control Center (NRIFACC) has conducted routine and systematic surveillance of RIFA annually. The comprehensive RIFA surveillance system maintained in Kinmen offers an exceptional opportunity for RIFA investigations across a range of temporal and spatial determinants.

The objective of this study was to assess the effects of land-use types, distance from the nearest road on different land-use types, and spatial factors on the risk of RIFA Successful Invasion or Remaining at the Highest level of invasion (RIFA SIRH for short) in Kinmen. To assess these effects, linear and nonlinear models were applied and compared. By understanding the mechanism of geographical expansions of RIFAs, this study provides insight into the invasion process of RIFAs and a range of control strategies.

## Methods and materials

### Study area

The main Kinmen Island is located between Taiwan and mainland China and consists of four townships: Jinsha, Jinhu, Jinning, and Jincheng (Fig. 1). Kinmen totals 132.17 km^2^, with a population of 127,202 people^17^. It lies in the subtropical monsoon region, with a southwest monsoon in the summer and a northeast monsoon in the winter. Between 2016 and 2019, the highest monthly mean temperatures varied from 30 to 33 °C from July to August, while the lowest mean temperature of 10 °C occurred from January to March^18^.

### Data

#### Land-use, land-use change, and road characteristic

Land-use data were obtained from The National Land Surveying and Mapping Center of the Ministry of the Interior in 2014 and in 2017. Land-use data in 2014 and 2017 were used as The Center surveyed land usages every 3–5 year, so these two years were the latest years when we conducted this study. We classified land-use data into four types: agricultural land, transportation usage (e.g., airports, roads, and harbors), natural land cover (e.g., forests and rivers), and artificial structures (e.g., buildings, schools, and recreation areas). In this study, to reflect land-use changes from 2014 to 2017, the variable of land-use from 2014 to 2017 in the models was divided into five categories: agricultural land, transportation usage, natural land cover, artificial structures, and land-use change. The area of each category was calculated by ArcGIS 10.7^19^.

Data for road characteristics were obtained from the 2016 traffic network map drawn by the Ministry of Transportation and Communications in Taiwan. The distance between a sampling tube and its nearest road was used in this study.

#### RIFA

**Figure 2:**
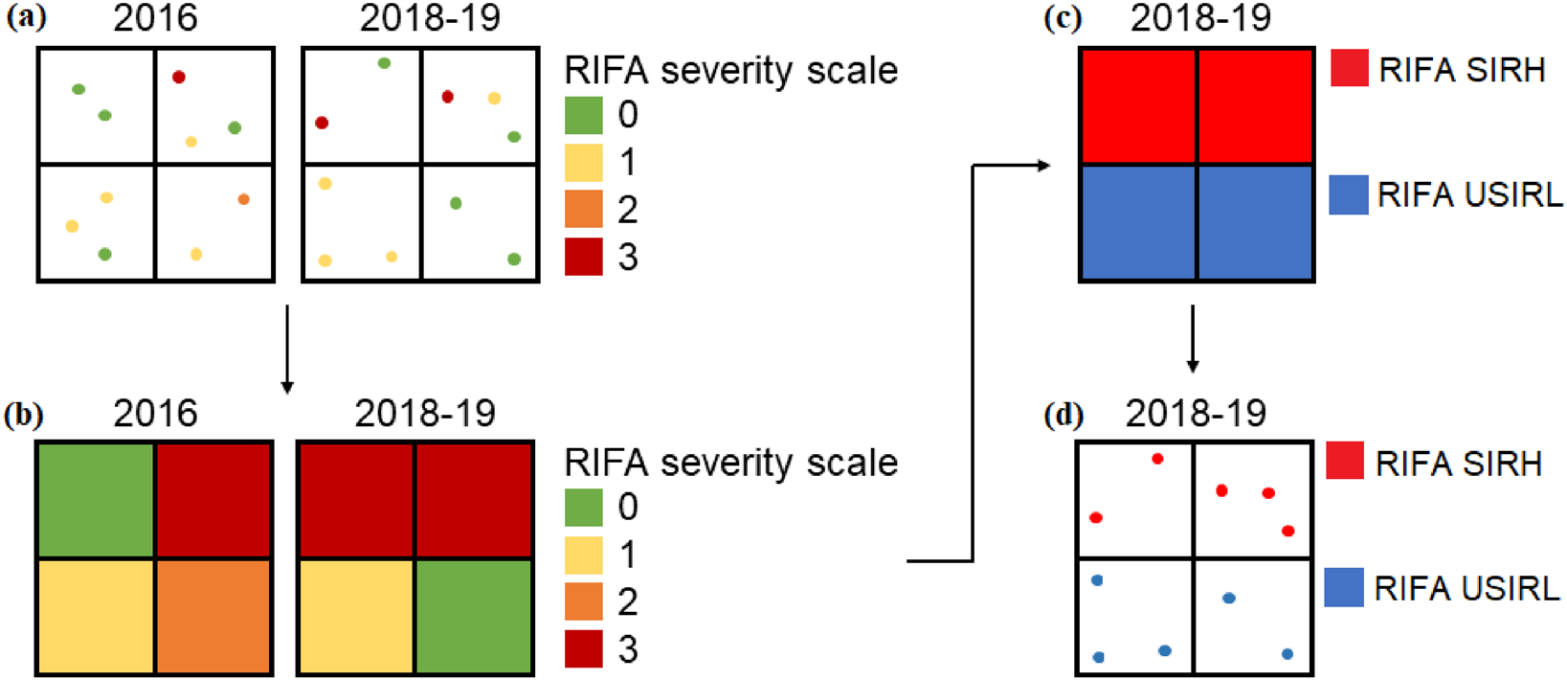
Quantification of RIFA successful invasion or remaining at the highest level of invasion (RIFA SIRH) and RIFA unsuccessful invasion or remaining at low levels of invasion (RIFA USIRL), 2018–2019.

The RIFA data were from the Taiwan RIFA surveillance system. The system included information of sampling tubes on geographic coordinates, date of collection and severity of RIFAs. The metric used for RIFA severity was taken a system designed by Harlan et al. as a reference^20^. Severity was divided into scales from scale 0 to scale 3: Scale 0 meant no RIFAs, scale 1 meant one to four RIFAs, scale 2 meant five to 49, and scale 3 meant more than 50 RIFAs were trapped in a sampling tube. Sampling tubes (12 cm long × 1.5 cm radius) contain potato chips that serve as bait and were placed in one or two tubes per 200 × 200 m^2^^21^. To detect the greatest severity scale in each grid, tubes were placed by specially trained RIFA surveillance personnel. However, as many reasons such as road constructions or changes in land-use, the locations of tubes might be slightly changed within the same grid in each year (Fig. 2a). Potato chips were used because they were available in the most supermarkets and cheap. In addition, the size of a chip was standardized.

As, on average, the effect of land-use change on the RIFA invasions would be 0.5–1 year lag for the development or establishment of a stable colony, we focused on RIFA spatial invasions in 2018–2019^11^. To quantify the RIFA invasions, RIFA data were extracted for the years 2016, 2018, and 2019. To symmetrically compare the spatial distributions of RIFA sampling tubes between 2016 and 2018–2019, Kinmen was divided into grids with dimensions of 200 m × 200 m. Each grid was marked with the scale value (i.e., 0, 1, 2, and 3) corresponding to the most severe sampling tube in that grid (Fig. 2a,b). Then, comparing the scales of marked grids in 2016 to those in 2018–2019, if the grids increased in scale or remained at scale 3, they were defined as RIFA SIRH (Fig. 2c). Then, the sampling tubes in the grids were assigned as RIFA SIRH in the period of 2018–2019 (Fig. 2d). If the grids with scales other than scale 3 were constant or if the grids decreased in scale between 2016 and 2018–2019 (Fig. 2b), they were defined as areas where RIFA were UnSuccessful Invasion or Remaining at Low levels of invasion (RIFA USIRL for short) (Fig. 2c). Afterwards, each tube was overlapped with land-use from 2014 to 2017 to extract its category for models.

#### Spatial factors of RIFA invasion

Geographical coordinates of RIFA sampling tubes were recorded based on the projected coordinate system (TWD97/TM2) in Taiwan to quantify the spatial locations. In the model, this variable represented the spatial effects on RIFA SIRH.

### Statistical models

We implemented three statistical models to compare the linear and nonlinear effects of covariates on RIFA SIRH. The first model was Generalized Linear Model (GLM). In GLM, the logit link function was used to capture the relationships of binary dependent variables (i.e., RIFA SIRH and RIFA UIRL) with independent variables. There were three independent variables: land use from 2014 to 2017, the distance between a sampling tube and its nearest road, and spatial factors.

Model 1: GLM

$$logit\left({y}_{i}=1\right)\left(\frac{P\left({y}_{i}=1\right)}{1-P\left({y}_{i}=1\right)}\right)\left(\frac{P\left({y}_{i}=1\right)}{P\left({y}_{i}=0\right)}\right) ={\beta }_{0}+ {\beta }_{1}{LanduseChange}_{i} + {\beta }_{2}{RoadDistance}_{i}+ {\beta }_{3}{Xcoord}_{i} + {\beta }_{4}{Ycoord}_{i} + {\beta }_{5}{Xcoord*Ycoord}_{i} + {\varepsilon }_{i}$$

where y_i_ represents RIFA SIRH of a specific grid where a sampling tube i falls in. We used the logit function as a link function for estimating the probability of RIFA SIRH. LanduseChange_i_ is a dummy variable referring to five categories in the land-use variable from 2014 to 2017 for tube i, RoadDistance_i_ is the distance between sampling tube i and the nearest road, Xcoord_i_ and Ycoord_i_ are the coordinates of sampling tube i, and ε is the residual.

To capture the nonlinear effects of covariates on RIFA invasion, the generalized additive model (GAM) was further applied.

Model 2: Generalized additive model (GAM-1)

$$logit({y}_{i}=1) (\frac{P({y}_{i}=1)}{1-P({y}_{i}=1)})(\frac{P({y}_{i}=1)}{P({y}_{i}=0)}) ={\beta }_{0} + {\beta }_{1}{LanduseChange}_{i} +{f}_{1}\left({RoadDistance}_{i}\right)+ {f}_{2}\left({Xcoord}_{i}{*Ycoord}_{i}\right) + {\varepsilon }_{i}$$

where dependent variable and LanduseChange_i_ variables are the same as Model 1. The f_1_ represents the smooth function of the distance between sampling tube i and the nearest road and the f_2_ is also a smooth function of $\left({Xcoord}_{i}{*Ycoord}_{i}\right)$ as a spatial factor to reflect geographical variations of the probability of RIFA SIRH.

Model 3: Generalized additive model with interaction (GAM-2)

$$logit\left({y}_{i}=1\right)\left(\frac{P\left({y}_{i}=1\right)}{1-P\left({y}_{i}=1\right)}\right)\left(\frac{P\left({y}_{i}=1\right)}{P\left({y}_{i}=0\right)}\right) ={\beta }_{0} + {\beta }_{1}{LanduseChange}_{i}+{f}_{1}\left({RoadDistance}_{i}\times {LanduseChange}_{i}\right)+ {f}_{2}\left({Xcoord}_{i}{\times Ycoord}_{i}\right) + {\varepsilon }_{i}$$

We added an interaction term (${RoadDistance}_{i}\times {LanduseChange}_{i}$) in Model 3 for reflecting the effect of different distances to the nearest road on RIFA SIRH under different land-use types. The f_1_ of the interaction term is the smooth function for representing non-linear effects on the probability of RIFA SIRH.

Three modes were compared by Akaike's information criterion (AIC) and ANOVA. All calculations were performed in R version 3.6.2^22^. The "stats" package was used for GLM, and the "mgcv" package was used for GAM^22–27^.

## Results

### Descriptive statistics

**Figure 3:**
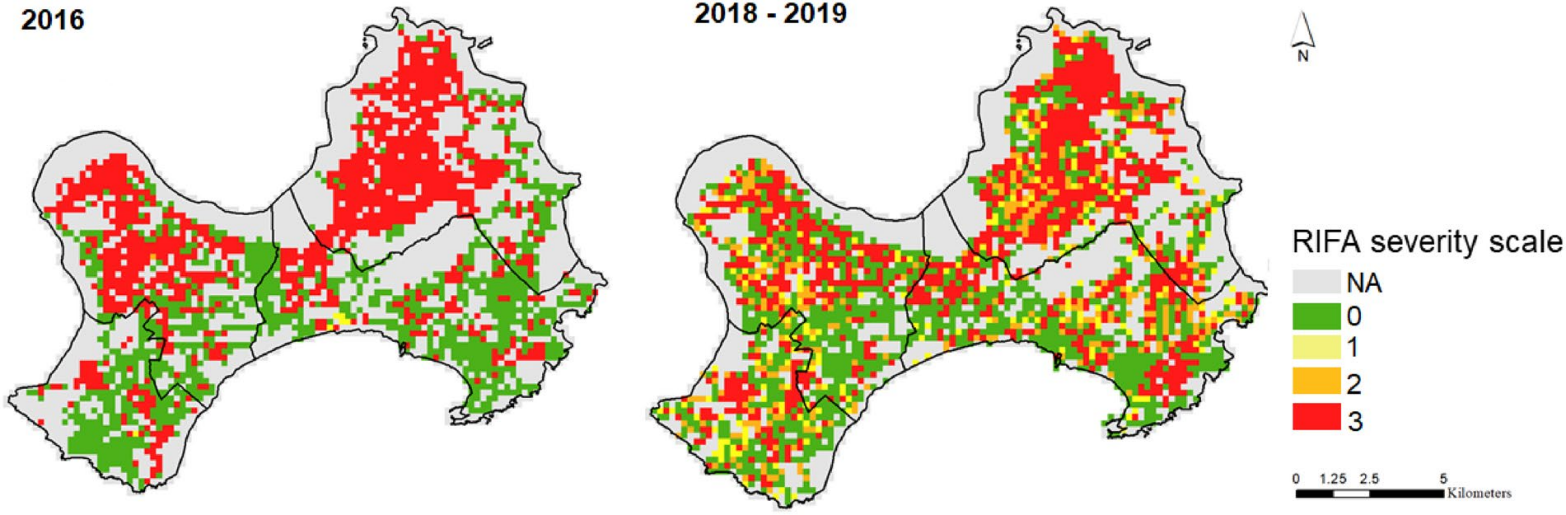
Distribution of RIFA severity in 2016 and 2018–2019. Areas with no sampling tubes were denoted as NA. This map is generated by ArcGIS 10.7^19^.

Overall, almost the same numbers of sampling tubes (N) were placed in 2016 (N = 6774) and 2018–2019 (N = 6918). In 2016, the majority of tubes had a severity of scale 3 (77.1%, N = 5220), followed by scale 0 (22.8%, N = 1542), scale 1 (0.1%, N = 8), and scale 2 (0.1%, N = 4) (Fig. 3). From 2018–2019, most tubes (55.2%, N = 3822) showed scale 0. The remaining sampling tubes were 6.9%, 10.2%, and 27.7% for scales 1, 2, and 3, respectively (Fig. 3).

**Figure 4:**
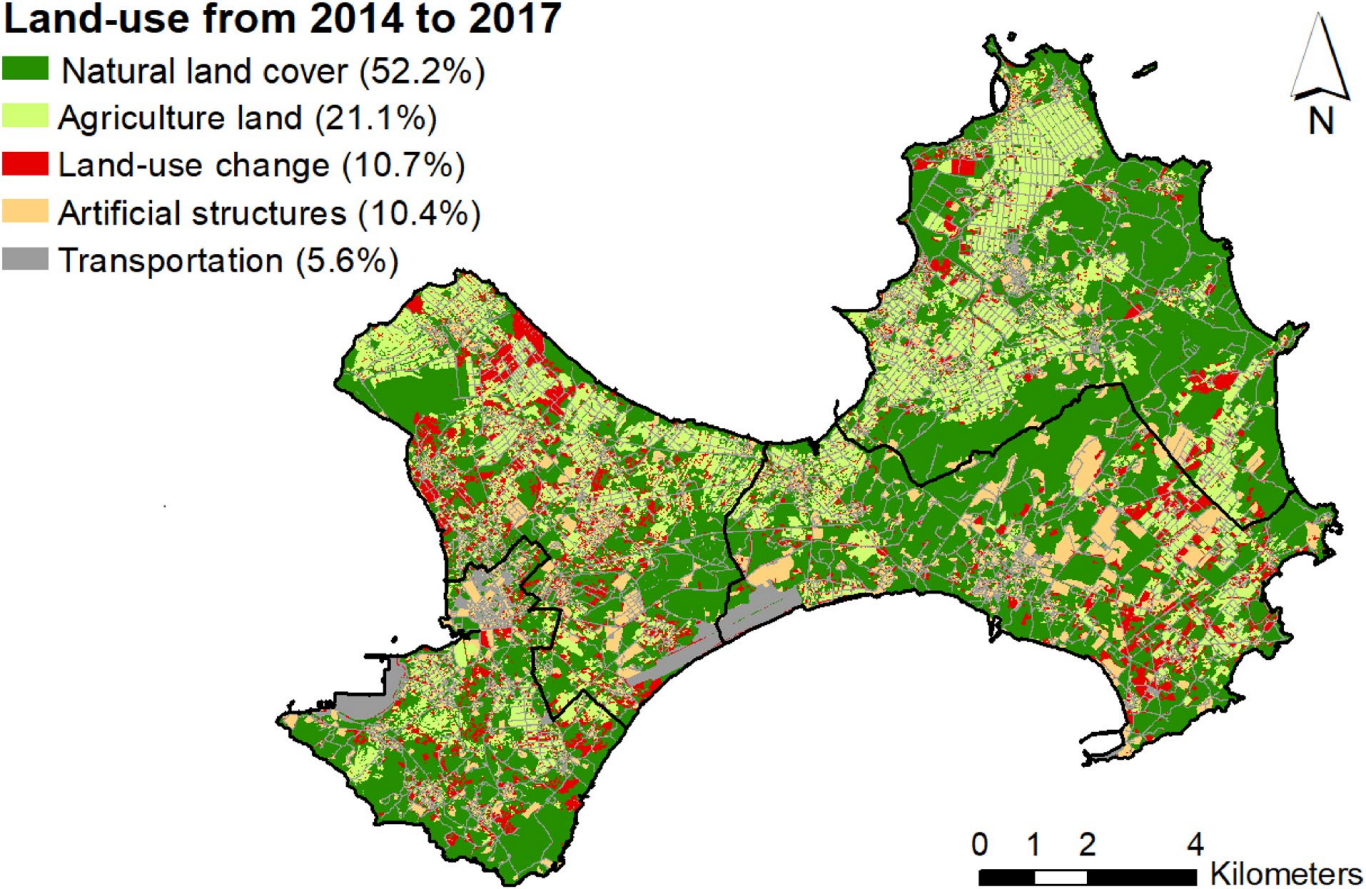
Spatial distribution of land-use change variable between 2014 and 2017. This map is generated by ArcGIS 10.7^19^.

For the land-use types from 2014 to 2017, the natural land cover covered more than half (52.2%, 69.0 km^2^) of Kinmen Island (Fig. 4). Agricultural land, artificial structures, and transportation covered 21.1%, 10.4%, and 5.6% of Kinmen, respectively. From 2014 to 2017, only 10.7% of the Kinmen area changed land-use types (Fig. 4). Regarding the distance between a sampling tube and its nearest road, the median from largest to smallest were 8.8 m, 2.9 m, 1.6 m, 1.2 m and 0 m for artificial structures, natural land cover, agricultural, land-use changes and transportation usages, respectively.

**Figure 5:**
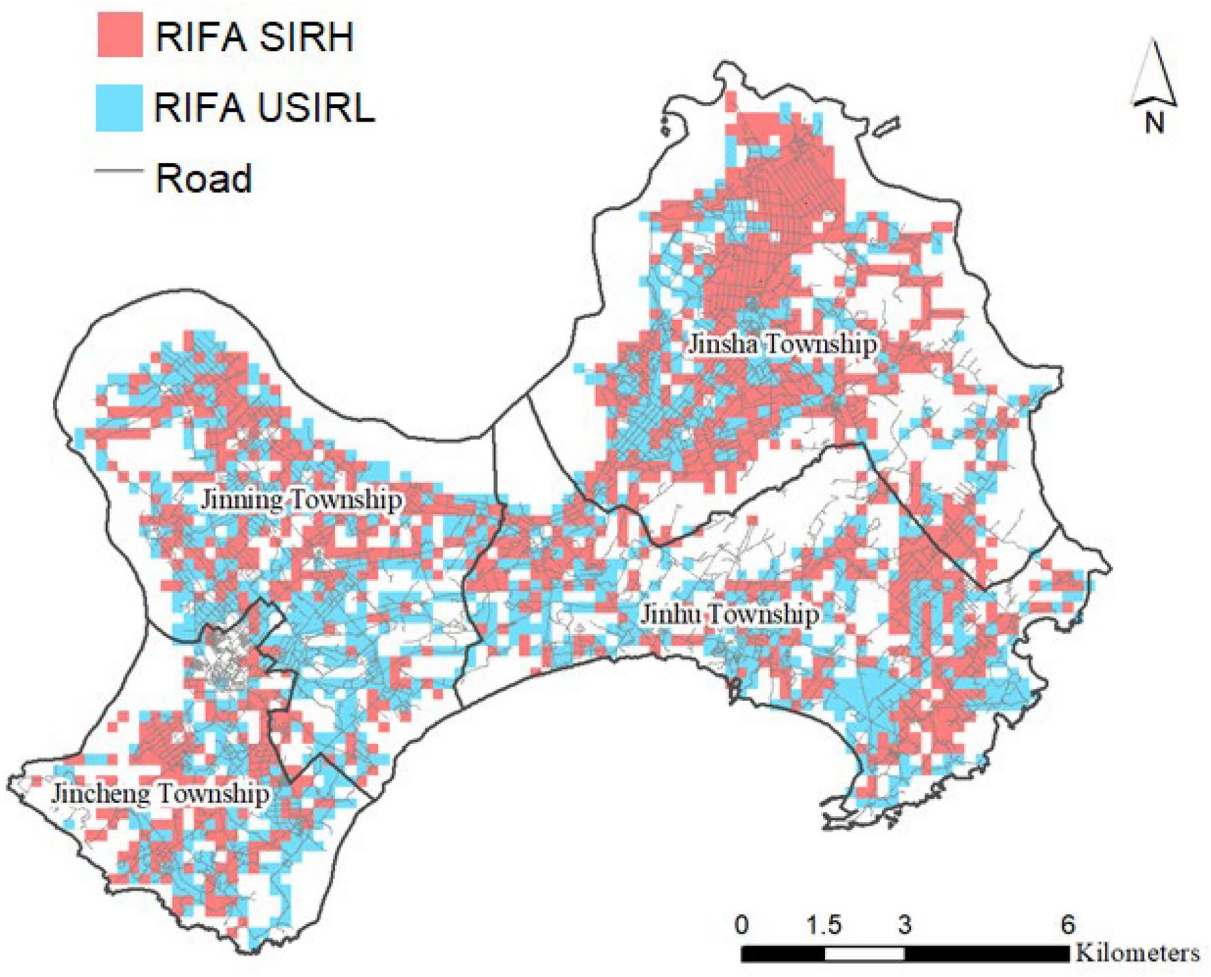
Spatial distribution of RIFA successful invasion or remaining at the highest level of invasion (RIFA SIRH) and RIFA unsuccessful invasion or remaining at low levels of invasion (RIFA USIRL), 2018–2019. This map is generated by ArcGIS 10.7^19^.

In terms of RIFA SIRH, spatial distributions of RIFA SIRH seemed to cluster in Jinsha and Jincheng townships (Fig. 5). There were over 50% of tubes were denoted as RIFA SIRH in each category of land-use type in 2018–2019 (Table 1). Comparing RIFA SIRH tubes to RIFA USIRL tubes, the land use from 2014 to 2017 was significantly associated with RIFA SIRH (chi-square = 168.7, p < 0.001).

**Table 1:** The distribution of sampling tubes with RIFA unsuccessful invasion or remaining at low levels of invasion (RIFA USIRL) and RIFA successful invasion or remaining at the highest level of invasion (RIFA SIRH), 2018–2019 (N = 6918).

Land-use from 2014 to 2017	Total number of sampling tubes (N)	RIFA USIRL	RIFA SIRH
		Number of sampling tubes	Number of sampling tubes
Transportation	1814 (100.0%)	668 (36.8%)	1146 (63.2%)
Agriculture land	1743 (100.0%)	372 (21.3%)	1371 (78.7%)
Natural land cover	1730 (100.0%)	714 (41.3%)	1016 (58.7%)
Land-use change	1193 (100.0%)	424 (35.5%)	769 (64.5%)
Artificial structures	438 (100.0%)	155 (35.4%)	283 (64.6%)

**Figure 6:**
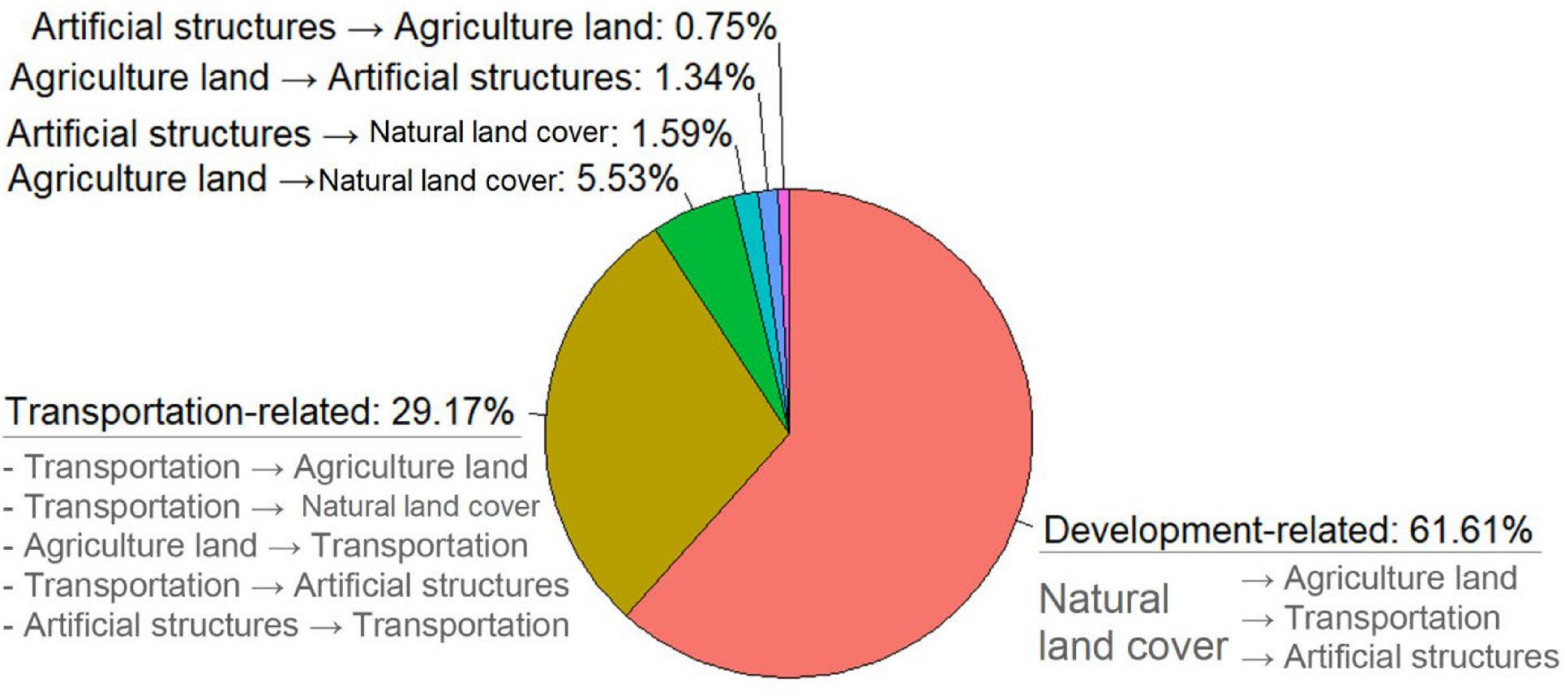
Percentage of RIFA sampling tubes in the category of land-use change from 2014 to 2017 (N = 1193). The land-use type before the arrow is the type in 2014. The land-use type after the arrow is the type in 2017.

The category of land-use change was further investigated. The number of tubes in the category of land-use change was 1193 in 2018–2019 (Table 1). Among the 1193 tubes, 61.6% were in development-related areas, which included natural land cover in 2014, while those areas were changed to agricultural lands, transportation usage, and artificial structures in 2017 (Fig. 6). In addition, 29.2% of tubes were on land used for transportation purposes either in 2014 or in 2017 (Fig. 6).

### Model results

The ANOVA test demonstrated that GAM-2 was a better fit than GAM-1 (df = 20.5, F value = 3.4, p value < 0.001), and GAM-1 was a better fit than the GLM (df = 24.6, F value = 18.5, p value < 0.001); GAM-2 thus had the best performance among the three models. A similar pattern was shown in AIC. The AICs for GLM, GAM-1 and GAM-2 were 8406.155, 8017.534, and 7996.118, respectively. GAM-2 had the lowest AIC, indicating that it was the best fit. Therefore, GAM-2 would be the focus for interpreting follow-up regression results.

The GAM model had both parametric part and nonparametric portions of the analysis. In the parametric results, based on the GAM-2 model (Table 2), the category of transportation usage had 1.3 times the odds of RIFA SIRH compared with natural land cover, significantly. Additionally, agricultural lands had 2.2 times greater odds of being invaded than natural land cover, significantly. Finally, the category of land-use change was 1.4 times more likely than natural land cover to have RIFA SIRH, significantly.

**Table 2:** GAM-2 regression model for RIFA successful invasion or remaining at the highest level of invasion in Kinman, Taiwan (N = 6,918).

Parameter	Parametric part			
	Odds ratio		z-value	p-value
	Estimation	Std Error		
(Intercept)	1.547	0.093	7.222	0.000*
Land-use from 2014 to 2017**				
Artificial structures	1.251	0.182	1.537	0.124
Transportation	1.346	0.134	2.980	0.003*
Agriculture land	2.247	0.203	8.942	0.000*
Land-use change	1.354	0.115	3.573	0.000*

Smooth terms	Nonparametric part			
	edf***	χ^2^	p-value	
f(RoadDistance) × natural land cover	4.992	11.718	0.067	
f(RoadDistance) × artificial structures	1.989	5.546	0.123	
f(RoadDistance) × transportation	3.157	13.362	0.006*	
f(RoadDistance) × agriculture land	3.430	8.756	0.081	
f(RoadDistance) × land-use change	2.377	10.719	0.013*	
f(Xcoord, Ycoord)	25.508	574.803	0.000*	

*Significant at α = 0.05.

**Natural land cover as the reference category.

***Effective degree of freedom.

**Figure 7:**
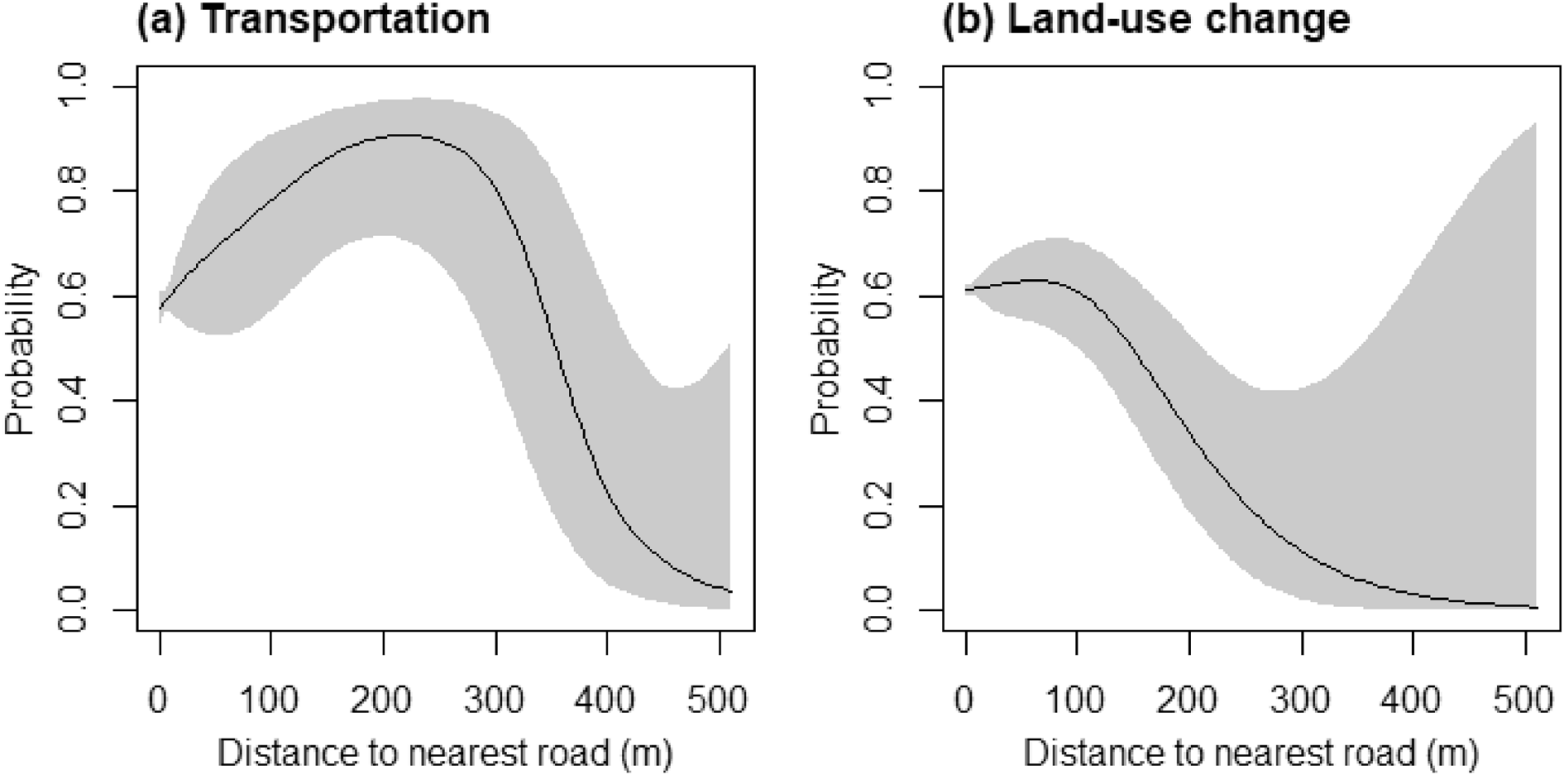
The non-linear associations between the distance to nearest road and (**a**) transportation (**b**) land-use change from 2014 to 2017. The x-axis is the distance of a sampling tube to the nearest road; the y-axis is the probability of RIFA successful invasion or remaining at the highest level of invasion.

The nonparametric part of the GAM-2 results contained variables that capture nonlinear impacts on RIFA SIRH. The interaction variable between distance to the nearest road and transportation usage was significantly associated with RIFA SIRH (Table 2). This result indicated that the category of transportation usage and the distance to the nearest road had significant nonlinear impacts on RIFA SIRH. The results further identified that approximately 60% of RIFA SIRH would occur in the 350 m stretch between the sampling tubes and the nearest roads (Fig. 7a). In the same vein, an interaction between distance from the nearest road and land-use change was significant (Table 2). It indicated that distance from the road to the areas where land-use change from 2014 to 2017 had a significant nonlinear association with RIFA SIRH. It was further showed that in the areas under land-use change, within 150 m from the nearest roads, the probability of RIFA SIRH was over 0.6 (Fig. 7b).

**Figure 8:**
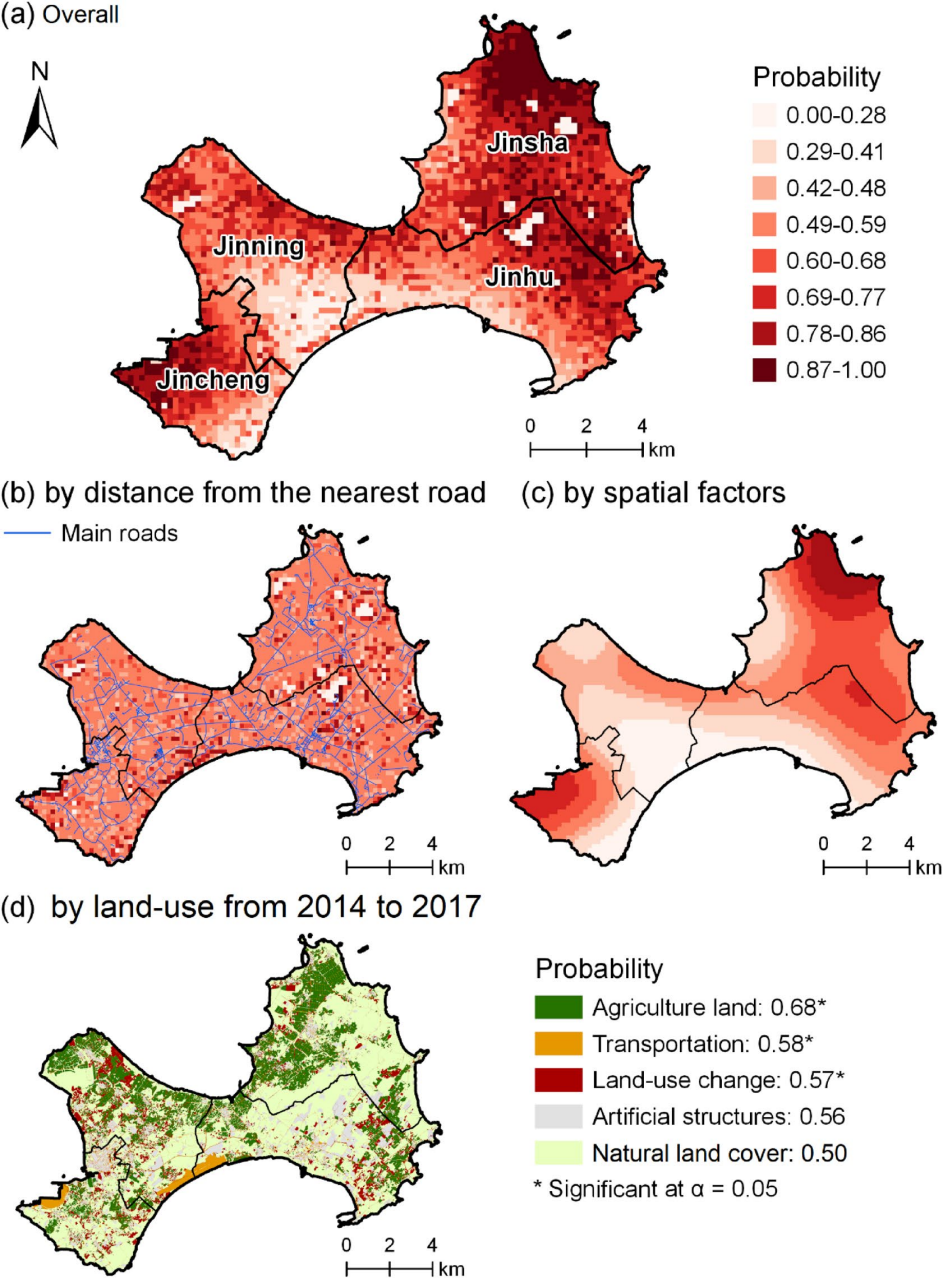
Risks of RIFA successful invasion or remaining at the highest level of invasion (RIFA SIRH) in Kinmen, 2018–2019, into (**a**) overall, (**b**) distance to nearest road, (**c**) spatial autocorrelation, and (**d**) land-use change from 2014 to 2017. This map is generated by ArcGIS 10.7^19^.

In summary, the overall risks of RIFA SIRH (Fig. 8a) are most affected by the following three components: the variable of distance from the nearest road (Fig. 8b), spatial factors (Fig. 8c), and land-use from 2014 to 2017 (Fig. 8d). In the land-use variable from 2014 to 2017, agricultural land, which was mainly distributed in Jinsha and Jinning townships (Fig. 8a), had the highest probability (i.e., 0.7) of RIFA SIRH (Fig. 8d). For the spatial factors (Fig. 8c), the highest risk areas were predicted in the northern (where RIFAs were first detected) and the western (Shuitou Pier) parts of the island (Fig. 1). As spatial factors were significant (chi-square = 574.8, p < 0.0001) in the nonparametric part (Table 2), the northern and western parts of Kinmen reflected significant risks of RIFA SIRH after adjusting for covariates.

## Discussion

This study used comprehensive surveillance data to profile RIFA invasions in time and space on an isolated island. By using this surveillance data, which were collected regularly together with information on land-use in different years, distinctions of RIFA severity can be compared, and RIFA SIRH were therefore identified. Our statistical model decomposed the spatial invasion risk into four geographic and anthropogenic factors: land-use characteristics, distances from RIFA sampling location to the nearest road, and spatial factors. For land use from 2014 to 2017, agricultural land, transportation usage, and land-use change had significantly higher odds of RIFA SIRH than natural land cover. Regarding the distance from the nearest road, RIFA invasions were most likely (> 60%) to occur within 350 m from the nearest road on the transportation usage land. Meanwhile, it was likely (> 60%) to have RIFA invasions within 150 m from the nearest road in areas where land-use change had occurred between 2014 and 2016. Finally, the highest risks of RIFA SIRH were identified around the pier area and the area of the earliest RIFA invasions on Kinmen. Our study provided an example showing how RIFA gradually expanded to the entire isolated island.

### Highest risks for agricultural land, transportation usage, and land-use change

#### Agricultural land

The vulnerability of agricultural lands to RIFA invasions has been reported in many studies. For example, a review by Apperson and Adams showed that RIFA often infested soybean fields in the United States^28^. Way and Khoo reviewed the RIFA infestation of crop plants, including sugar cane and cotton^29^, and indicated that crop invasion by RIFAs was a common occurrence. The study conducted by Stuhler et al. demonstrated that in unthinned patches, RIFA mounds were likely to occur in agricultural lands compared to woodlands in South Carolina^30^. Thus, the results of our study align with the literature in finding that agricultural land tends to be highly assailable by RIFAs.

The large majority of agricultural lands on Kinmen Island include sorghum farms, peanut farms, and other food crop farms^31^. These farms need to be plowed or cultivated at least twice per year. Therefore, soil disturbances by humans could be the reason for the defenselessness against RIFA invasions. The potential mechanism is that soil disturbances destroy habitats for all living organisms, including RIFA. However, RIFAs reestablished their colonies faster than others^30,32^. Thus, RIFAs became one of the dominant species in highly disturbed areas. Higher soil disturbances associated with higher RIFA abundances were evidenced by the study by Stuhler et al.^30^ in which the authors compared the thinned areas to unthinned areas, identifying more RIFA mounds in thinned plots. King and Tschinkel also conducted a field experiment on different levels of soil disturbances. They demonstrated that higher numbers of RIFAs persisted at higher levels of disturbance (i.e., plowing) than at lower levels (i.e., mowing)^32^.

#### Land for transportation usage

The land-use type for transportation purposes, including roads and ports (i.e., seaports and airports), was also identified as a risk factor for RIFA SIRH in this study (Table 2). Among the 1814 sampling tubes in the transportation area, there were 1768 sampling tubes for roads and 46 for ports. As most of the sampling tubes were set along roads in the present study, it could be deduced that roadsides or road cuts were at risk of being infested by RIFA. This result was in compliance with previous studies in the U.S., showing that areas beside roads such as roadsides and road margins provided suitable habitats for RIFA development^11,33–37^.

Roadsides or road cuts had significant risks of RIFA SIRH in Kinmen, which could be due to frequent disturbances from vehicles. In Kinmen, most roads have only one lane or two narrow lanes. When two vehicles traveling in opposite directions pass each other, they will sometimes take turns or pull over onto the side, resulting in frequent soil disturbance. Roadsides or areas near roads are generally considered highly disturbed^10,11,34,38^, and narrow and disturbed areas suitable for RIFA establishment were demonstrated by Stiles and Jones^12^.

In addition to disturbances along roads, some vehicles may also transport RIFAs in potted plants and soil. Newly-mated queens may potentially attach to the surface of vehicles and fall during transportation, further facilitating invasions near roadsides. This traffic-related dispersal process has been documented in many plant species^39–41^.

Road maintenance could also be a reason for the high risks near roadsides. Road maintenance involves moving soil from one place and adding soil to construction sites. If the transported soil is contaminated by RIFAs, the maintenance areas will likely be occupied by RIFA. A case report by King et al. revealed how RIFA spread to roadsides by road maintenance^32^.

Ports, in addition to roads, are another land type for transportation usages. Our finding was in line with previous studies showing that airports or seaports were common areas of RIFA invasion in Taiwan and neighboring countries. For example, Taoyuan International Airport was considered one of the earliest RIFA infestation locations in Taiwan^42,43^. RIFAs were also detected in container yards in Taiwan’s Kaohsiung commercial port in 2018^44^. In other Asia–Pacific countries, such as China, South Korea, Japan, and Australia, RIFAs have also been reported at ports in the last decade^44,45^.

Ports in this study consist of one seaport and one airport (Fig. 1). Based on the predicted risk of RIFA SIRH (Fig. 8a), one of the highest risk areas was around Shuitou Pier in Jincheng township (Fig. 1). The Pier area had high risks could be because it is one of the cargo container entrances on Kinmen Island. Shipping cargo containers have been suggested to facilitate the movement of RIFAs from abroad or between domestic ports^42–44^. Container yards can become infested when RIFA-contaminated cargo containers are unloaded^44,46^. In addition to possible contributions from cargos, the pier area had high risks of invasions, which could be due to environmental conditions. This can be supported by the risk of spatial factors, showing that the Pier area had high risks (Fig. 8c). One of the possible environmental factors could be that floating rubbish tends to accumulate in the Pier area^47^. Studies have shown that nonnative species, including ants, can travel with marine litter to new locations^32,48–51^.

The Kinmen Shangyi Airport is the other cargo entrance in Kinmen (Fig. 1). Intuitionally, because of cargo containers, the airport area was expected to have risks similar to those in the pier area; however, the risks of RIFA invasions in the airport area were considerably lower (Fig. 8a). The differences in risks could be due to their cargo carrying capacities. In 2018, the airport had 6778 tons of cargo, but the pier had one million tons of cargo^52,53^. Differences in the types of cargo between the two locations may also play a role in invasion risks. From 2001 to 2018, the majority of goods arriving at the Pier included building stones and block stones from China^53^. These products have higher risks of being contaminated by RIFAs than goods such as ferrous articles and eggs arriving from the airport of Taiwan^53,54^.

#### Land-use change

The land-use change category was identified as a risk factor for RIFA SIRH in the current study. Among land-use change areas, 61.6% were natural land cover in 2014 but were converted to agricultural land, transportation areas, and artificial structures in 2017, which we designated development-related areas (Fig. 6).

As previously mentioned, the reasons why the land-use change category had a high risk of RIFA invasion could be due to anthropogenic disturbances. Taking development-related areas as an example, when natural land cover such as forests are changed to other land usages, the first step may be to remove vegetation by clearcutting or plowing. These activities involve soil or habitat disturbances and could aid in the establishment of RIFA populations^55^. Then, if lands are changed to build houses or schools (i.e., artificial structures), soil disturbances could also occur during construction activities^56^. For lands that are changed to transportation usages, moving and adding RIFA-contaminated soil could occur during road construction.

### Effects of roads on RIFA SIRH

Distances to the nearest roads were important for understanding invasion where undergoing land-use change, as well in places used as transportation lands (Fig. 7). These land-use categories share a common feature: roads. Meanwhile, agriculture lands had the greatest level of RIFA SIRH, but did not show interaction with distance to roads (Table 2). This could be because agriculture lands were far from roads as compared to land-use change and transportation lands. The median distances to roads from these three land-use categories supported this speculation. Therefore, from this study, it can be deduced that the roads could play a role to transport RIFAs to areas closer to road (i.e., land-use change and transportation). However, the effects of roads on RIFA SIRH did not appear when the areas away from roads (i.e., agricultural lands).

### Lowest risk in natural land cover

In the present study, natural land cover were identified as the lowest risk category of RIFA SIRH among the five land-use categories (Fig. 8d). This finding was in line with the study conducted by Brown et al., showing that a high percentage of canopy cover was associated with a low mean number of RIFAs in Texas between 2008 and 2010^57^. In addition, Tschinkel and King investigated longleaf pine forests in Florida in 2012 and found that RIFA had difficulty establishing long-term colonies in the forest^35^. However, in another longleaf pine forest in Georgia, the ant survey conducted by Stuble et al. revealed that RIFAs were the predominant species in the ant community from 2006 to 2007^58^. Wetlands also had high numbers of RIFAs. In northern Florida, Tschinkel observed that RIFA mounds clustered near pond margins^11^.

Natural land cover in Kinmen had the lowest risk of RIFA invasions, which could be because most areas (> 75%, data not shown) are forests. The forests are preserved and protected by the Forestry Bureau of Taiwan. Because of protection, forests can avoid most anthropogenic disturbances, such as soil excavation, which are known as one of the factors facilitating RIFA relocation^32,59,60^. Additionally, the forest environment is cool, humid, and shaded, which are unfavorable environmental conditions for RIFAs^1,12,30,34,61,62^.

### Implications of study findings for RIFA management in Kinmen

#### Public communications

To date, the Kinmen County Animal and Plant Disease Control Center (KAPCDC) has launched a program aimed at raising public awareness of RIFAs on the island through newspapers, social media, and posters. In addition, for RIFA control, the KAPCDC has listed certified pesticides such as pyriproxyfen and lambda-cyhalothrin for the use of controlling RIFAs on agricultural lands. Nevertheless, our study documented that a greater risk of RIFA invasions still occurred on agricultural lands and lands used for transportation, suggesting communications should target owners of agricultural lands as well as the general public in future campaigns. Many individuals of the general public may not be able to identify ant species, so communications should therefore emphasize the importance of reporting any ant mounds, especially along roads. As different sociodemographic groups react to source information differently, communications have to be tailored to ages and educational levels^7^. For example, for students in primary school, the study by Madeira et al. showed that by teaching activities including insect specimens and short-film presentations, students increased their awareness of the importance of pest control^63^. For owners of agricultural lands and workers at ports, educational activities on basic RIFA knowledge and pesticide treatments with suitable communication methods may be needed. Those methods included regular face-to-face discussions on RIFA elimination strategies in the meetings of farmers’ associations or a system sharing updated materials likely to be contaminated with RIFAs^64,65^.

#### RIFA control personnel

To prioritize resources, according to the findings from this study, we suggest that government staff focus on the controls within 350 m from the nearest road on transportation usage land and within 150 m from the nearest road on the areas where land-use change occurred between 2014 and 2016. The authorities could consider integrated pest management approaches, which include chemical and biological controls, to preserve the local ecosystem^66^.

For agricultural lands, RIFA management mainly relies on awareness and reports from owners, as control personnel cannot perform inspections and intervention on private agricultural lands without the owners’ permissions, Although control personnel cannot directly perform interventions on private land, plant quarantine officers in seaports, which were a high-risk area in this study, can prevent RIFA importation by checking cargos to ensure that RIFAs are not stowaways on materials such as plants, rocks, and soil.

## Limitation

The distributions of RIFAs could be influenced by many abiotic and biotic factors, but not all of them were considered in this study. For example, one of the abiotic factors is control activities. On Kinmen, RIFA control has been carried out by the KAPCDC since 2017, but control efforts were not taken into account in this study. This was because we did not have sufficient information on the intervention locations. Biotic factors such as predators and coexisting ants were not considered as well^34^.

Natural dispersals were also not assessed in this study. RIFAs can be naturally spread by nuptial flights. However, the effects of nuptial flights may be small, as the study by King et al. suggested that compared to human-mediated transportation, nuptial flights were a small driver in the dispersion of polygyne RIFAs^32^. In addition to nuptial flights, natural disturbances such as typhoons can distribute RIFAs. During the study period from 2016 to 2019, Kinmen was hit by a typhoon in 2016. This may have influenced the results, as the typhoon event occurred before RIFA data collection.

The datasets we used from different periods could be a problem for the findings. Land-used data for road from 2016 as this year was the only available year during the study period. It may not present the road characteristics such as road construction after 2017. In addition, the findings of RIFA invasions could be underestimated if areas were changed their usages again in 2018–2019. However, if land-use types did not change in 2018–2019, our findings were likely to reflect the RIFA situation as, on average, the effect of land-use change on the RIFA invasions would be 0.5–1 year lag for the development or establishment of a stable colony^11^.

The other limitation of our design is tubes share the same severity in each 200 × 200 m^2^ grid (Fig. 2d), but each tube may not have the same severity scale in each grid (Fig. 2a). As we have different locations of sampling tubes between years, our approach can systematically compare severities between years. Thus, we think our design is a trade-off between the land-use resolution and the ability to relate delta severity. To establish a better surveillance system for RIFA, the authorities are suggested to use the same locations of sampling location tubes in the future.

## Data Availability

The datasets used and/or analyzed during the current study available from the corresponding author on reasonable request.
